# Circulatory cathepsin K as biomarkers in older adults with sarcopenia: a case-control study

**DOI:** 10.3389/fmed.2025.1654694

**Published:** 2025-09-10

**Authors:** Huaqing Liu, Hongdou Liu, Yan Xing, Gengze Wang, Jialin Wang, Ye Fan, Peiwen Zhang, Shangxin Wang, Hu Zhang

**Affiliations:** ^1^Department of Gastroenterology, Nanyang Central Hospital, Nanyang, Henan, China; ^2^Department of Cardiovascular Surgery, Nanyang Central Hospital, Nanyang, Henan, China; ^3^Department of Endoscopy, Nanyang Central Hospital, Nanyang, Henan, China

**Keywords:** cathepsin D, cathepsin K, sarcopenia, older adults, skeletal muscle

## Abstract

**Background:**

This study explores the relationship between circulating cathepsin K (CatK) and cathepsin D (CatD) levels and sarcopenia in older adults.

**Methods:**

This case-control study included 343 participants aged more than 65 from Nanyang Central Hospital. Sarcopenia was diagnosed using AWGS criteria, requiring low handgrip strength (HGS) and reduced appendicular skeletal muscle mass (ASM). Fasting blood samples were collected to measure CatD and CatK levels via ELISA. The study compared these levels between groups and evaluated their diagnostic value using ROC curve analysis.

**Results:**

Serum CatK levels were significantly higher in participants with low HGS, low ASM, and sarcopenia (all *p* < 0.05). CatK negatively correlated with HGS (β = −0.899, *p* = 0.016) and showed diagnostic value with an AUROC of 0.704 for sarcopenia. CatD levels showed no significant differences or correlations. The optimal CatK cutoff for sarcopenia was 5.53 ng/mL, with high CatK associated with increased odds of low HGS (OR = 1.895, *p* = 0.014) and sarcopenia (OR = 3.926, *p* < 0.001).

**Conclusion:**

Circulating CatK is a promising biomarker for sarcopenia, offering potential for early diagnosis and therapeutic targeting.

## Introduction

Sarcopenia, a progressive and multifactorial syndrome characterized by a decline in skeletal muscle mass and function, poses a significant challenge to the health of older adults (1). Its prevalence increases with age and is associated with frailty, falls, mobility limitations, and a higher risk of mortality (2, 3). This condition not only diminishes physical capacity but also reduces the quality of life, necessitating a deeper understanding of its pathophysiology to identify potential biomarkers and therapeutic targets for better clinical management (4–6). Traditional risk factors such as age, malnutrition, physical inactivity, and lifestyle have been implicated in the development of sarcopenia (7, 8). However, emerging research suggests that biochemical and molecular markers may offer more specific insights into the condition's progression and pathogenesis.

Cathepsins are lysosomal proteases that play a key role in intracellular protein degradation and turnover, and they have been increasingly recognized for their involvement in various age-related disorders (9, 10). Among these, cathepsin D (CatD) and cathepsin K (CatK) have distinct roles in proteolytic signaling (11–14). CatD, an aspartic protease, is primarily secreted in response to oxidative stress and has been associated with various conditions involving tissue remodeling and inflammation (15). Elevated levels of CatD have been reported in the context of heart failure, where it is believed to contribute to adverse outcomes by influencing cellular stress responses and autophagy (16, 17).

Similarly, CatK, a cysteine protease, is known for its robust collagenolytic and elastolytic activities, and it has been implicated in tissue remodeling processes, particularly in the context of injury and inflammation (18). Recent studies have shown that CatK expression is upregulated in skeletal muscle following cardiotoxin-induced injury, contributing to muscle fibrosis and loss of functional muscle mass (19). Moreover, CatK may directly involve in muscle fibrosis via IRS1 (20). This suggests that CatK may play a pivotal role in the muscle remodeling and repair processes that are often disrupted in sarcopenia (20). Given these findings, both CatK and CatD are potential candidates as biomarkers for conditions involving muscle loss and dysfunction, including sarcopenia.

Despite the growing recognition of cathepsins in muscle-related disorders, their specific roles and potential as biomarkers in sarcopenia remain underexplored. In this case-control study, we aimed to investigate the relationship between circulatory CatK and CatD levels and the occurrence of sarcopenia in older adults. Our objective was to determine if these cathepsins could serve as reliable biomarkers for sarcopenia, thereby aiding in early diagnosis and therapeutic intervention.

## Methods

### Populations

This case-control study was conducted at the Department of Gastroenterology, Nanyang Central Hospital, in accordance with the Declaration of Helsinki and was approved by the hospital's Ethics Committee (ID: 20190771). All participants provided written informed consent, and their data were anonymised. From May 2019 to May 2024, eligible older individuals aged 65 and above, who provided written consent, were recruited. However, individuals with malignancies or incomplete data on sarcopenia and relevant biomarkers were excluded to maintain the study's integrity and focus.

### Data collection

Comprehensive baseline information for each participant was meticulously gathered through a thorough review of their electronic medical records maintained by our institution. Key data elements included demographic information such as age and sex, detailed anthropometric measures like height and weight, and a comprehensive medical history covering smoking and alcohol habits as well as comorbid conditions. Clinical assessments encompassing electrocardiograms and chest X-rays were also documented. To quantify the burden of comorbidities, the well-established Charlson Comorbidity Index (CCI) was employed (21). Upon admission, a series of laboratory tests were performed to measure critical biomarkers, including red blood cell count (RBC), hemoglobin (Hb), blood glucose (GLU), and albumin (ALB) levels, providing a comprehensive baseline health profile for each participant.

### Cathepsin measurement

Fasting venous blood samples were collected from participants on the morning following admission. These samples were utilized to evaluate two key serum proteases—Cathepsin D (CatD) and Cathepsin K (CatK). The assessment was performed using specific ELISA kits: a human CatD ELISA kit (JL12469-96T, Jonlnbio) and a human CatK ELISA kit (JL11425-96T, Jonlnbio), following the manufacturers' instructions. After careful blood sample processing, the collected sera were applied to microplates pre-coated with capture antibodies specific to CatD and CatK. Following incubation and thorough washing, the bound analytes were detected via HRP-conjugated secondary antibodies and a chromogenic substrate. The absorbance was measured spectrophotometrically, and the concentrations of CatD and CatK were determined by interpolation from a calibration curve constructed using serial dilutions of known standards.

### Sarcopenia assessment

Sarcopenia diagnosis followed the Asian Working Group for Sarcopenia (AWGS) guidelines (22, 23), requiring both low handgrip strength (HGS) and reduced appendicular skeletal muscle mass (ASM). Specifically, HGS was deemed low if below 28 kg in men or 18 kg in women. ASM was insufficient if below 7.00 kg/m^2^ in men or 5.70 kg/m^2^ in women. HGS was measured thrice with a calibrated spring-loaded dynamometer, and the peak value was recorded. For ASM evaluation, bioimpedance analysis (BIA) was performed using the InBody BWA2.0 device. The device captured bioimpedance at eight frequencies (1 kHz to 3 MHz) across four body segments: right and left upper and lower limbs. Its algorithm then automatically calculated ASM and normalized it by squaring the individual's height, enabling accurate participant comparisons.

### Statistical analysis

Continuous variables are presented as mean ± standard deviation, and categorical variables as counts with percentages. Normally distributed continuous variables were analyzed with independent Student's *t*-tests, while non-parametric data were analyzed with Wilcoxon rank-sum tests. Normality was examined with the Shapiro–Wilk test; non-normally distributed variables (CatD and CatK) were analyzed with the Wilcoxon rank-sum tests. Categorical variables were assessed via Chi-squared or Fisher's exact tests. Receiver operating characteristic (ROC) curves evaluated the relationship between CatD/CatK and sarcopenia, determining optimal cutoffs via the Youden index. Patients were grouped based on these cutoffs, and sarcopenia traits were compared between groups. Linear and logistic regression models investigated the associations of CatD/CatK with HGS, ASM, and sarcopenia, adjusting for relevant covariates. A *P*-value below 0.05 was considered statistically significant. All analyses were conducted using R software, version 4.1.2.

## Results

### Populations

**Figure 1:**
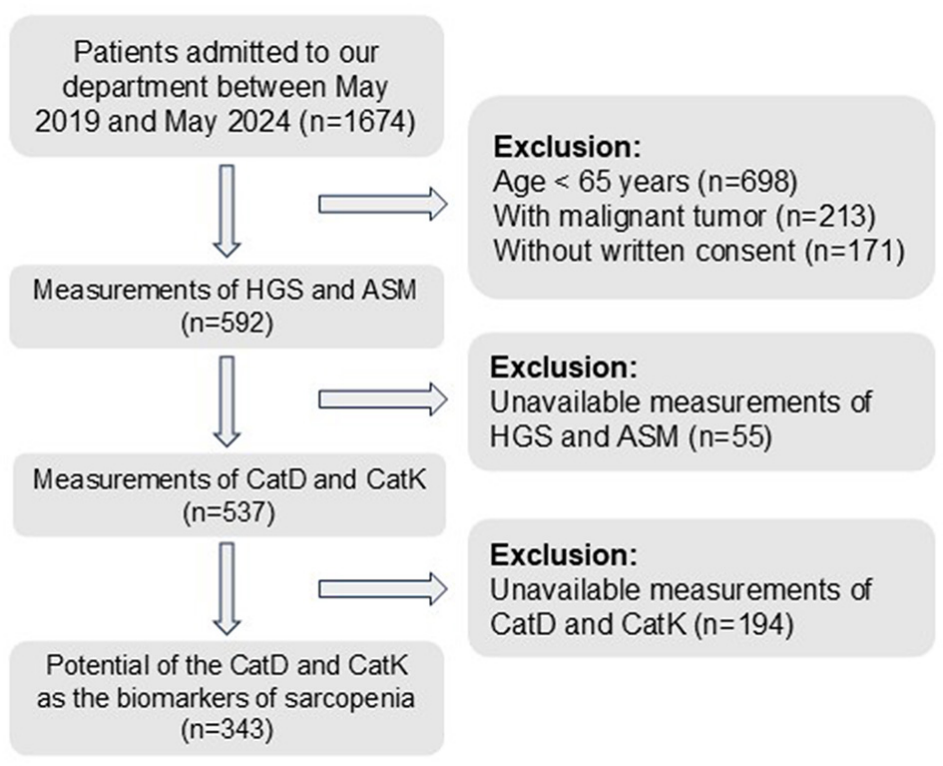
Flow chart of our study.

After applying the inclusion and exclusion criteria and measuring the relevant markers and sarcopenia characteristics, this study included 343 patients, with 49 diagnosed with sarcopenia (Figure 1). In the normal group (*n* = 294), the mean age was 73.41 ± 6.71 years, the mean BMI was 20.69 ± 5.34 kg/m^2^, and 159 patients (54.08%) were female. In the sarcopenia group (*n* = 49), the mean age was 73.06 ± 6.39 years, the mean BMI was 21.25 ± 5.48 kg/m^2^, and 17 patients (34.69%) were female. Baseline characteristics showed no significant differences between the two groups in terms of age, BMI, and several other variables (all *p* > 0.05, as shown in Table 1). However, the distribution of females was significantly higher in the normal group compared with the sarcopenia group (*p* = 0.012).

**Table 1:** Baseline features of the individuals included in this study.

**Variables**	**Overall**	**Normal**	**Sarcopenia**	**p value**
	**(*****n*** = **343)**	**(*****n*** = **294)**	**(*****n*** = **49)**	
Age (years)	73.36 ± 6.66	73.41 ± 6.71	73.06 ± 6.39	0.81
BMI (kg/m^2^)	20.77 ± 5.36	20.69 ± 5.34	21.25 ± 5.48	0.317
Sex (female)	176 (51.31%)	159 (54.08%)	17 (34.69%)	0.012
Smoking history (yes)	100 (29.15%)	90 (30.61%)	10 (20.41%)	0.146
Alcoholism history (yes)	68 (19.83%)	58 (19.73%)	10 (20.41%)	0.912
CCI score (>4)	112 (32.65%)	96 (32.65%)	16 (32.65%)	>0.999
Electrocardiogram (abnormal)	93 (27.11%)	80 (27.21%)	13 (26.53%)	0.921
Chest radiograph (abnormal)	82 (23.91%)	73 (24.83%)	9 (18.37%)	0.326
Hypertension (yes)	124 (36.15%)	108 (36.73%)	16 (32.65%)	0.582
RBC (10^∧^12/L)	4.26 ± 1.22	4.28 ± 1.21	4.18 ± 1.29	0.515
Hb (g/L)	90.06 ± 26.14	90.43 ± 26.17	87.83 ± 26.06	0.503
ALB (g/L)	36.83 ± 12.25	36.83 ± 12.31	36.83 ± 12.01	0.774
GLU (mmol/L)	5.22 ± 1.56	5.24 ± 1.57	5.13 ± 1.53	0.696
CatD (pg/mL)	97.22 ± 54.50	96.70 ± 54.60	100.34 ± 54.36	0.448
CatK (ng/mL)	4.62 ± 1.86	4.40 ± 1.73	5.90 ± 2.08	<0.001

BMI, Body Mass Index; CCI, Charlson Comorbidity Index; RBC, Red Blood Cell; GLU, Blood Glucose; Hb, Hemoglobin; ALB, Albumin; CatD, Cathepsin D; CatK, Cathepsin K.

### CatD and CatK levels

**Figure 2:**
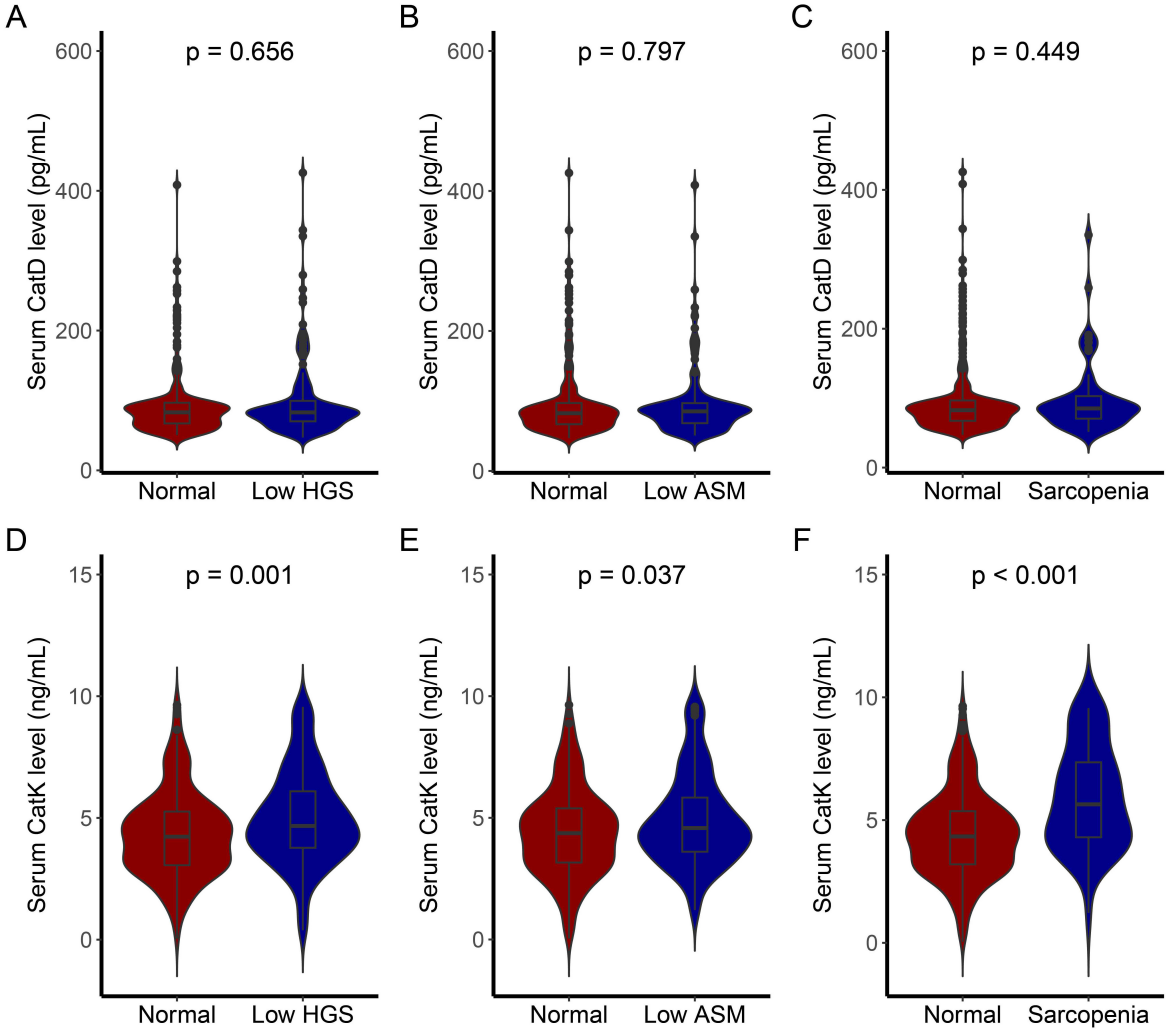
CatD and CatK levels of individuals with different sarcopenia traits. **(A)** Comparison of CatD levels between individuals with low HGS and normal individuals; **(B)** Comparison of CatD levels between individuals with low ASM and normal individuals; **(C)** Comparison of CatD levels between individuals with sarcopenia and normal individuals; **(D)** Comparison of CatK levels between individuals with low HGS and normal individuals; **(E)** Comparison of CatK levels between individuals with low ASM and normal individuals; **(F)** Comparison of CatK levels between individuals with sarcopenia and normal individuals.

Based on the AWGS criteria, patients with low HGS, low ASM, and sarcopenia were identified. Comparisons of serum CatD and CatK levels among these groups and the normal cohort revealed the following (Figure 2) the low HGS group had comparable CatD levels to the normal group (*p* = 0.656), but significantly higher CatK levels (*p* = 0.001). Similarly, the low ASM group showed no significant difference in CatD levels (*p* = 0.797) but elevated CatK levels (*p* = 0.037). In the sarcopenia group, CatD levels were similar to the normal group (*p* = 0.449), but CatK levels were substantially higher (*p* < 0.001), indicating a notable increase in CatK among sarcopenic patients.

To investigate the associations between these markers and HGS/ASM further, linear regression analyses were performed. Univariate models were first built with the markers as variables, followed by multivariate models adjusted for age, sex, and BMI. As presented in Table 2, CatK exhibited a negative correlation with HGS in both univariate (β = −0.933, *p* = 0.012) and multivariate (β = −0.899, *p* = 0.016) analyses. For ASM, CatK showed negative correlations in both univariate (β = −1.43, *p* = 0.063) and multivariate (β = −1.43, *p* = 0.061) models, although these did not reach statistical significance. CatD did not show significant correlations with either HGS or ASM in the analyses conducted.

**Table 2:** Univariate and multivariate linear models of CatD and CatK for HGS and ASM.

**Variables**	**HGS**				**ASM**			
	**Univariate**		**Multivariate**		**Univariate**		**Multivariate**	
	β	***p***	β	***p***	β	***p***	β	***p***
CatD (continuous)	−0.011	0.393	−0.009	0.481	0.001	0.862	0.001	0.887
CatK (continuous)	−0.933	0.012	−0.899	0.016	−0.143	0.063	−0.143	0.061

HGS, Hand Grip Strength; ASM, Appendicular Skeletal Muscle Mass; CatD, Cathepsin D; CatK, Cathepsin K.

### Diagnostic values of CatD and CatK

**Figure 3:**
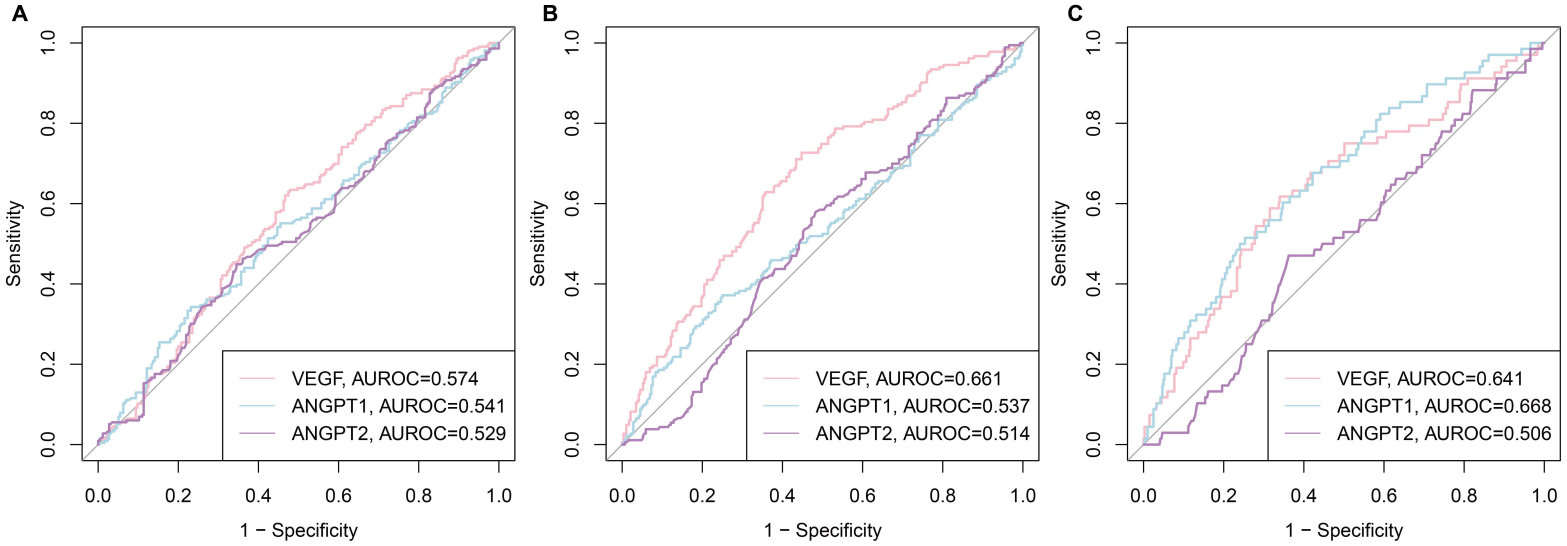
ROC curves of CatD and CatK levels for low HGS, low ASM, and sarcopenia. **(A)** for HGS; **(B)** for ASM; **(C)** sarcopenia.

The diagnostic utility of CatD and CatK for sarcopenia traits was evaluated via ROC curve analysis for low HGS, low ASM, and sarcopenia. Figure 3 displays the ROC curves for CatD and CatK in predicting these conditions. CatK demonstrated better predictive performance than CatD across all three conditions. For low HGS, CatK had an AUROC of 0.607 vs. CatD's 0.486. For low ASM, the AUROCs were 0.567 for CatK and 0.508 for CatD. In predicting sarcopenia, CatK achieved an AUROC of 0.704, while CatD had an AUROC of 0.534. Optimal cutoff values of CatD and CatK for sarcopenia were determined using the Youden index: 82.48 pg/mL for CatD and 5.53 ng/mL for CatK. Patients were categorized into subgroups based on these thresholds to facilitate further analysis.

### Relationships between CatK and sarcopenia

The relationships between CatD, CatK, and sarcopenia were explored by comparing the sarcopenia characteristics among different groups based on the cutoff values of these two cathepsins (Table 3). For CatD, the high CatD group exhibited similar HGS, ASM, and sarcopenia proportion to the normal CatD group (all *p* > 0.05). For CatK, the high CatK group showed significantly higher proportions of low HGS (54.65% vs. 38.52%, *p* = 0.009) and sarcopenia (29.07% vs. 9.34%, *p* < 0.001) compared to the normal CatK group, while similar HGS (24.99 ± 12.43 vs. 27.23 ± 12.92, *p* = 0.162), ASM (6.77 ± 2.44 vs. 7.34 ± 2.69, *p* = 0.083), and similar proportion of low ASM (48.84 vs. 37.35%, *p* = 0.06).

**Table 3:** Sarcopenia traits of individuals grouped by CatD and CatK.

**Outcomes**	**Overall**	**Normal CatD**	**High CatD**	***p***	**Normal CatK**	**High CatK**	***p***
	**(*n* = 343)**	**(*n* = 164)**	**(*n* = 179)**		**(*n* = 257)**	**(*n* = 86)**	
HGS (kg)	26.66 ± 12.82	27.37 ± 13.23	26.02 ± 12.44	0.329	27.23 ± 12.92	24.99 ± 12.43	0.162
ASM (kg/m^2^)	7.20 ± 2.64	7.34 ± 2.69	7.06 ± 2.59	0.328	7.34 ± 2.69	6.77 ± 2.44	0.083
Low HGS	146 (42.57%)	69 (42.07%)	77 (43.02%)	0.86	99 (38.52%)	47 (54.65%)	0.009
Low ASM	138 (40.23%)	61 (37.20%)	77 (43.02%)	0.272	96 (37.35%)	42 (48.84%)	0.06
Sarcopenia	49 (14.29%)	19 (11.59%)	30 (16.76%)	0.171	24 (9.34%)	25 (29.07%)	<0.001

HGS, Hand Grip Strength; ASM, Appendicular Skeletal Muscle Mass; CatD, Cathepsin D; CatK, Cathepsin K.

To control for potential confounding effects of covariates on the cathepsins, univariate and multivariate logistic regression analyses were performed (Table 4). Multivariate models were adjusted for age, sex, and BMI. The results showed that high CatK was significantly associated with low HGS [OR = 1.895, 95% CI (1.142, 3.163), *p* = 0.014] and sarcopenia [OR = 3.926, 95% CI (2.080, 7.449), *p* < 0.001]. Continuous CatK was also significantly associated with low HGS [OR = 1.222, 95% CI (1.081, 1.387), *p* = 0.002], low ASM [OR = 1.158, 95% CI (1.025, 1.312), *p* = 0.019], and sarcopenia [OR = 1.515, 95% CI (1.282, 1.798), *p* < 0.001]. Both high CatD and continuous CatD showed no significant associations with low HGS [OR = 1.001, 95% CI (0.997, 1.005), *p* = 0.621], low ASM [OR = 0.998, 95% CI (0.994, 1.002), *p* = 0.444] or sarcopenia [OR = 1.000, 95% CI (0.995, 1.005), *p* = 0.986].

**Table 4:** Univariate and multivariate logistic models of CatD and CatK for sarcopenia traits.

**Variables**	**Low HGS**		**Low ASM**		**Sarcopenia**	
	**OR [95 %CI]**	***p***	**OR [95 %CI]**	***p***	**OR [95 %CI]**	***p***
**Univariate**						
CatD (continuous)	1.002 [0.998, 1.006]	0.369	0.999 [0.995, 1.003]	0.657	1.001 [0.995, 1.006]	0.665
CatK (continuous)	1.227 [1.090, 1.387]	0.001	1.160 [1.032, 1.308]	0.014	1.520 [1.292, 1.802]	<0.001
High CatD	1.039 [0.677, 1.597]	0.86	1.275 [0.827, 1.970]	0.272	1.537 [0.834, 2.893]	0.173
High CatK	1.923 [1.176, 3.162]	0.009	1.601 [0.977, 2.623]	0.061	3.979 [2.125, 7.487]	<0.001
**Multivariate**						
CatD (continuous)	1.001 [0.997, 1.005]	0.621	0.998 [0.994, 1.002]	0.441	1.000 [0.995, 1.005]	0.865
CatK (continuous)	1.222 [1.081, 1.387]	0.002	1.158 [1.025, 1.312]	0.019	1.512 [1.282, 1.798]	<0.001
High CatD	0.976 [0.627, 1.521]	0.916	1.197 [0.764, 1.879]	0.432	1.484 [0.799, 2.812]	0.216
High CatK	1.895 [1.142, 3.163]	0.014	1.587 [0.951, 2.652]	0.077	3.926 [2.080, 7.449]	<0.001

HGS, Hand Grip Strength; ASM, Appendicular Skeletal Muscle Mass; CatD, Cathepsin D; CatK, Cathepsin K.

## Discussion

This study delved into the potential roles of circulatory CatK and CatD as biomarkers in older adults with sarcopenia. Our findings unveiled that serum CatK levels were markedly elevated in individuals with low HGS, ASM, and overall sarcopenia, as per the AWGS criteria. Notably, CatK demonstrated a negative correlation with HGS and showed diagnostic value in predicting sarcopenia, with a higher area under the receiver operating characteristic (AUROC) curve compared to CatD. In contrast, CatD levels did not exhibit significant differences between the sarcopenia and normal groups, nor did they show meaningful associations with HGS or ASM. These results spotlight CatK as a promising biomarker for sarcopenia, potentially aiding in early diagnosis and guiding therapeutic strategies.

The pathophysiology of sarcopenia is intricate, involving an interplay of proteolytic signaling pathways (6, 24, 25). Cathepsins, as lysosomal proteases, are pivotal in protein degradation and turnover (26). CatK, a cysteine protease with potent collagenolytic and elastolytic activities, has been implicated in tissue remodeling processes (26, 27). Previous studies have indicated that CatK expression is upregulated in skeletal muscle following injury, contributing to muscle fibrosis and functional loss (19, 20). Our study aligns with these findings, suggesting that the elevated CatK levels in sarcopenic patients might reflect its role in disrupted muscle remodeling and repair processes. The negative correlation between CatK and HGS further reinforces the notion that CatK could be a key player in the muscle wasting aspect of sarcopenia (20).

On the other hand, CatD, an aspartic protease primarily secreted in response to oxidative stress, has been associated with conditions involving tissue remodeling and inflammation (28). However, in our study, CatD failed to show significant associations with sarcopenia-related traits. This discrepancy might be attributed to differences in the pathophysiological mechanisms underlying various muscle-related disorders (25, 29). While CatD may play a role in certain inflammatory and remodeling processes, its involvement in the specific context of sarcopenia might be less pronounced or more complex than that of CatK (30, 31).

The diagnostic utility analysis via ROC curves provided further evidence of CatK's superiority over CatD as a sarcopenia biomarker. The determined optimal cutoff values for CatK could serve as practical thresholds for identifying individuals at risk of sarcopenia. This is particularly valuable given the often silent progression of sarcopenia in older adults, where early detection is crucial for implementing timely interventions to mitigate its debilitating consequences. From a translational perspective, the AUROC of 0.704 for CatK approaches that of established sarcopenia biomarkers (32). Given the low cost and wide availability of ELISA, CatK could be integrated into routine geriatric assessment pathways, especially where access to imaging is limited.

Our study, however, is not without limitations. The relatively small sample size and single-center design may limit the generalizability of our findings. Future large-scale, multi-center studies are warranted to validate the diagnostic accuracy and clinical relevance of CatK in diverse populations. Additionally, the cross-sectional nature of this study precludes definitive conclusions about the causality between CatK levels and sarcopenia progression. Longitudinal studies tracking CatK levels and sarcopenia outcomes over time would provide more robust insights into their dynamic relationship. Lastly, because the study was observational and relied on consecutive enrolment, no prospective power analysis was undertaken; the relatively small number of sarcopenia cases (*n* = 49) may therefore limit statistical power and generalizability.

In conclusion, this study highlights the potential of circulatory CatK as a biomarker for sarcopenia in older adults. Its diagnostic value and associations with key sarcopenia traits underscore the need for further exploration of CatK-targeted therapeutic strategies. As research on sarcopenia continues to evolve, CatK may emerge as a critical factor in the early identification and management of this geriatric syndrome, ultimately contributing to improved quality of life for older individuals.

## Data Availability

The raw data supporting the conclusions of this article will be made available by the authors, without undue reservation.
